# Physiotherapy in Parkinson's Disease: Building ParkinsonNet in Czechia

**DOI:** 10.1155/2017/8921932

**Published:** 2017-05-22

**Authors:** Ota Gal, Martin Srp, Romana Konvalinkova, Martina Hoskovcova, Vaclav Capek, Jan Roth, Evzen Ruzicka

**Affiliations:** ^1^Department of Neurology and Centre of Clinical Neuroscience, First Faculty of Medicine and General University Hospital, Charles University in Prague, Katerinska 30, 128 21 Prague, Czech Republic; ^2^Applied Neurosciences and Brain Imaging, National Institute of Mental Health, Topolova 748, 250 67 Klecany, Czech Republic

## Abstract

**Objective:**

We conducted a questionnaire survey to investigate the availability and quality of physiotherapy (PT) for Parkinson's disease (PD).

**Background:**

Despite evidence about the benefits of PT, there is no data regarding its use in Czechia.

**Methods:**

Questionnaires were sent to 368 PD patients seen in a single movement disorders centre within two years (inclusion criteria: idiopathic PD, Hoehn and Yahr stage <5, and residence in Prague) and to 211 physical therapists (PTs) registered in Prague. The patient questionnaire evaluated limitations in 6 core areas and in activities of daily living and inquired about experience with PT. The PTs questionnaire evaluated knowledge about PD, number of PD patients treated yearly, and details of therapy.

**Results:**

Questionnaires were returned by 248 patients and 157 PTs. PT was prescribed to 70/248 patients. The effects were satisfactory in 79% and lasted >3 months in 60/64. About half of the PTs have no experience with PD patients, 26% reported <3, and 5% see >10 yearly. The most widely used techniques were neurodevelopmental treatments.

**Conclusion:**

Present PD healthcare model in Czechia is suboptimal (low PT prescription, non-evidence-based PT). Implementation of European PT Guidelines for PD and the introduction of an efficient model of care are needed.

## 1. Introduction

The prevalence of Parkinson's disease (PD) is increasing [1, 2], paralleled by growing healthcare expenses [3]. PD is a complex disorder, for which interprofessional care is appropriate [3–6]. Despite increasing evidence about the benefits of physiotherapy [7–15], detailed insight into the current provision of physiotherapy, as well as barriers and facilitators for optimal care, is lacking in Czechia. However, the Dutch model for Community Healthcare is available and has proven both being cost effective and providing greater patient satisfaction [16, 17]. The authors of this model have developed stepwise recommendations for the application of such model in other countries [3]. As the first step they recommend gaining insight into current healthcare, that is, patient utilization, and satisfaction with provided allied healthcare as well as allied healthcare provider's expertise and volume of PD patients treated. Moreover, this healthcare model is recommended by the European Physiotherapy Guideline for PD (EPGPD) [9], which is binding for Czechia, and the Czech Union of Physical therapist (UNIFY) took part in their development. Consequently, we conducted a questionnaire survey to investigate the quality and availability of physiotherapy for PD patients in Czechia based on previously published studies [18, 19].

## 2. Methods

Questionnaires approved by the Ethics Committee of General University Hospital in Prague were sent to 368 PD patients seen in April 2013–April 2015 in our department. Inclusion criteria were idiopathic PD, Hoehn and Yahr stage <5, and residence in Prague. The questionnaire evaluated the patients' limitations in activities of daily living (PADLS) [20], frequency of falls, limitations in 6 core areas (gait, transfers, manual dexterity, stability and falls, posture, and physical condition), their relative importance to the patient, and the patient's motivation to improve in them. Patients, who were referred to a physiotherapist (PT) because of PD, were also asked about the specifics of the therapy. Finally they were asked to estimate the time willing to travel to a PT specialized in PD.

A second questionnaire was delivered to 211 PTs working in a central district of Prague (Prague 2). PTs were first asked to provide details on their involvement in the healthcare system, years of experience, knowledge, interest, and education in PD treatment. Questions related to the PTs' knowledge about PD treatment addressed physiotherapy, occupational therapy, speech therapy, nursing, and neurological care. Consequently the questionnaire evaluated number of PD patients treated yearly, most frequent reasons for referral and medical specialization of the referring physician. Also, PTs were asked to provide opinion on the rightfulness of referral, on the importance of individual core areas, on the quality of communication in their team, and on most important barriers in the improvement of PD specific healthcare in Czechia. Details on the provided physiotherapy were required.

Responses from both questionnaires were, due to their nature, analysed using nonparametric statistical methods. Ordinal variables were compared among groups using a Mann-Whitney *U* test in case of comparisons of two groups and using a Kruskal-Wallis ANOVA in case of more than two groups. Categorical variables were subject to contingency tables and a Fisher exact test of independence in contingency tables. For expressing a relation of two ordinal variables, a Spearman correlation coefficient was evaluated. Moreover, a factor and cluster analyses were performed; details of these two methods are given in a text further. *p* values less than 0.05 were considered as statistically significant. Analyses were conducted using R statistical package, version 3.2.3.

## 3. Results

**Figure 1:**
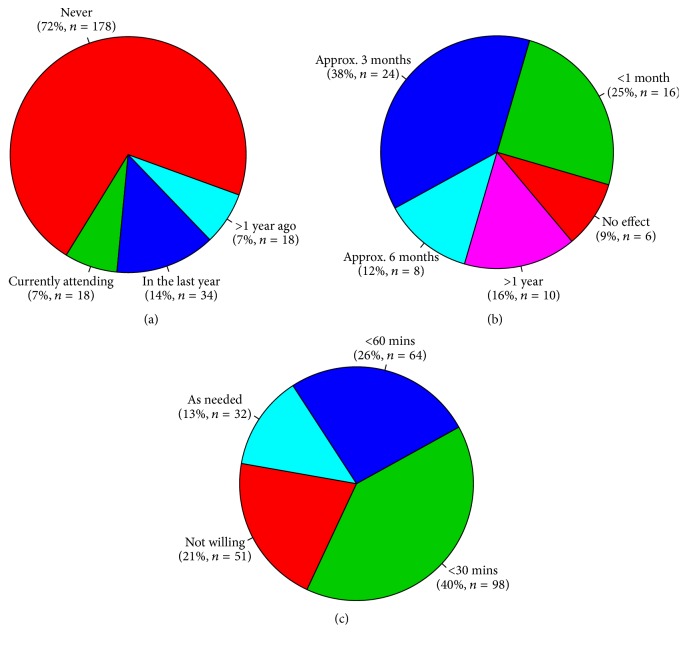
(a) Experience with PD specific physiotherapy (*n* = 248). (b) Duration of the effect of physiotherapy (*n* = 64). (c) Time patients are willing to travel to PT (*n* = 245).

Questionnaires were returned by 248 patients and 157 PTs. Not all questionnaires were completely filled, so the sample size differs in some questions. Patients reported no or mild difficulties in ADL in 59% (147/248) and a high level of difficulties or extreme difficulties in 19% (47/248). Nearly 20% (48/247) were repeat fallers and 30% (73/247) very frequent fallers [21], while 38% (94/247) reported no falls in the last year. Impairment in all core areas, as well as the motivation to improve, referral to physiotherapy because of the respective core areas, and the marked average order of importance of the core areas are given in Table 1. Only 28% of the patients had experience with physiotherapy prescribed because of PD (Figure 1(a)). These patients reported at least partial satisfaction with the quality of explanation of the possibilities of physiotherapy by the referring physician in nearly 96% and sufficient satisfaction with the explanation by a PT in nearly 60%. The quality of explanation by physicians and by PTs was the same (*p* = 0.078, Spearman's correlation coefficient *ρ* = 0.413, *p* = 0.000). On a subjective scale from 0 (no benefit) to 10 (maximal benefit) the median of the effect of the therapy was 5 (IQR 3–7) with the effect that lasted less than one month in 25%, 3 months in 38%, 6 months in 12%, and one year in 16%. In 9% the patients felt no effect (Figure 1(b)). The overall expected duration of the effect was 4.9 months. The patients' expectations were rather or completely met in 79%. Mean of the time patients are willing to spend travelling to a PT specialist was estimated to be 36 minutes (for details see Figure 1(c)).

A factor analysis was used to analyse all answers related to impairment in core areas, answers related to patients' will to improve and the number of falls in the last year. The main goal of a factor analysis is to reveal and calculate hidden or not directly measured factor/factors that influence behaviour of the subjects. The analysis was performed with an Ordinary Least Squares factoring method and a Varimax rotation. It showed that the patients' answers were determined by two factors, actual impairment and will to improve. These factors explain 57% of the variability of the data. The will to travel far to a PD specialized PT diminishes with severity of impairment (*p* = 0.000) but increases with will to improve (*p* = 0.000). We also showed correlation of time willing to spend travelling to a PT specialist with PADLS (*ρ* = −0.372, *p* = 0.000). Patients who had high level of difficulties or extreme difficulties in ADL were willing to travel 13 minutes, while those with no or moderate difficulties were willing to travel 41 minutes.

**Figure 2:**
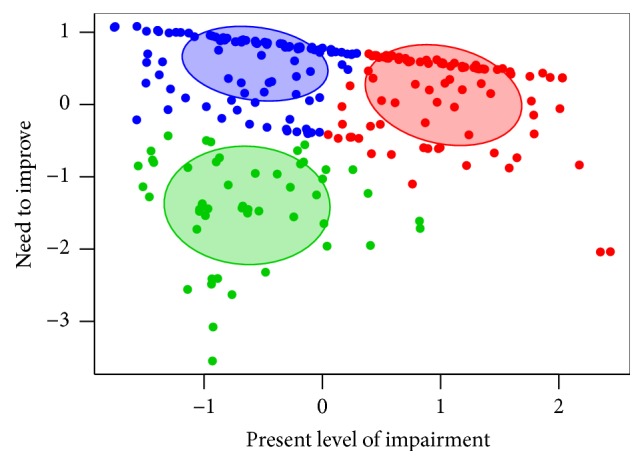


A cluster analysis was performed to find and describe groups of patients that are somehow similar in their actual impairment and will to improve. We used a *K*-Means Clustering method and showed that our patients could be divided into three not particularly distinct groups: (1) patients with both above average impairment and will to improve, (2) patients with below average impairment but above average will to improve, and (3) patients with both below average impairment and will to improve (Figure 2).

PTs that filled the second questionnaire were in 58% involved in hospital care, were relatively equally distributed in groups according to years of experience, and claimed to have interest in PD in 70%. This interest depends on number of PD patients treated yearly (*p* = 0.003) but does not depend on the length of PTs' practice (*p* = 0.310) or their knowledge about PD (*p* = 0.262). In 93% they did not attend any PD specialized course. PTs marked their knowledge about physiotherapy in PD as substandard in 25%, standard in 63%, and above standard in 13%. The situation in case of PD specific occupational therapy, speech therapy, nursing, and neurological care was similar, but slightly more shifted towards substandard knowledge. Generally, knowledge about these forms of PD care strongly correlated with knowledge about physiotherapy in PD (*p* = 0.000 in all cases). The attendance of a PD specific physiotherapy course did not correlate with increased knowledge about PD (*p* = 0.362). PTs reported in 52% that they see no PD patients per year and in 26% less than 3 and only 5% of them take care of more than 10. The mean estimate of PD patients treated yearly was for this group 2.63. The number of PD patients treated yearly correlated with knowledge about PD (*p* = 0.000). Those PTs in our group who treat at least 3 PD patients yearly had significantly greater interest in PD (*p* = 0.003). PTs reported similar referral rates from neurologists, rehabilitation physicians, and geriatrists (*p* = 0.073). Most frequent reasons for referral and the average order of importance of the core areas for patients are given in Table 1. PTs do not value all core areas the same (*p* = 0.000). The comparison of the order of importance of the core areas as reported by PTs, PD patients, and the referring physicians (based on the reported reason of referral; see Table 1) showed significant differences in priorities of all three groups (*p* = 0.000).

PTs found the communication in their team rather adequate or adequate in 53% and 31% of them marked the small amount of PD patients treated yearly as the most important barrier in the improvement of PD specific healthcare in the Czech Republic, followed by insufficient communication between healthcare professionals in 24%. The median of the length of therapy provided was 30 minutes (IQR 30–45) and the median of the total number of therapy sessions was eight (IQR 6.25–10). The length and total number of therapy did not correlate with PD specific physiotherapy course attendance (*p* = 0.438 and *p* = 0.882, resp.). Neither did they correlate with the PTs' knowledge about physiotherapy in PD (*p* = 0.544 and *p* = 0.723, resp.). PTs work with PD patients individually in 82% which correlated neither with knowledge about physiotherapy in PD (*p* = 1.000), nor with PD specific physiotherapy course attendance (*p* = 0.400). The most widely used physiotherapy techniques were neurodevelopmental treatments (NDTs) like the Bobath concept or Proprioceptive neuromuscular facilitation in 20%, followed by gait training (in 11%) and soft tissue therapy (in 10%). Techniques used by PTs were not related to knowledge about PD specific physiotherapy (*p* = 0.063).

## 4. Discussion

The main results of our study show low physiotherapy prescription rate, small number of patients treated yearly by PTs, discrepancy among PTs, PD patients, and the referring physicians in prioritizing core areas, and use of non-evidence-based physiotherapy techniques and finally that there are no patients with above average impairment but below average motivation. Other results show interesting findings about correlations among different PTs' characteristics (interest, length of practice, knowledge, and PD specific physiotherapy course attendance) and therapy parameters (length and number of therapy sessions, effect duration, and used techniques) and finally about most important barriers for optimal care and about communication among healthcare professionals and PD patients.

Results showing low prescription rate of physiotherapy need interpretation. One could object that not all patients in our group needed physiotherapy, that is, those who did not perceive any problem or those who did not want to improve. In order to gain insight into the prescription rate in case of patients who were both impaired and motivated, we adopted the concept of a patient-relevant problem [19]. The prescription rate in this subgroup (i.e., in those who reported impairment in a core area and declared interest in improvement) ranged between 15 and 22% which is comparable to the rate in the whole group of patients (14–21%). We did not find any dependence of prescription on current impairment in case of gait (*p* = 0.358), manual dexterity (*p* = 0.068), posture (*p* = 0.116), and physical condition (*p* = 0.128) and on the patient's motivation in all core areas (*p* values ranging from 0.088 to 0.638). Such prescription rate is lower than those reported in previous studies [19, 22, 23]. The chance for receiving physiotherapy for the three most important core areas for PD patients (gait, transfers, balance, and falls) is nearly three times lower than it was in Netherlands in 2009 [19]. The prescription rate for instability and falls was only approximately 18% in our study group although 62% of the patients reported at least one fall in the last year and PD patients are four times more endangered by hip fractures than healthy controls [24].

Those of our patients who received physiotherapy (median 30 minutes, 8x) reported high effect with expectations met or rather met in 79% and with a surprisingly long duration estimate (4.9 months). This seems to be overestimated when compared to available data from systematic reviews and meta-analyses of various physiotherapy modalities (resistance and aerobic training), which report lower or similar effect duration but with much longer and frequent therapy sessions [11, 25–27]. On the other hand the follow-up examination in these studies was performed exactly after 3 or 6 months, respectively, so the effect could have lasted longer here as well. A careful interpretation of our data is thus necessary as the reported duration was not tested objectively and patients may overstate duration of the effect.

The reported quality of the explanation of the possibilities of physiotherapy by the referring physician might be viewed as sufficient. On the other hand, sufficient explanation by a PT should be a matter of course, and this was reported only in 60%. It might therefore be considered relatively low especially because insufficient communication about therapy was described as a barrier in adherence to physiotherapy and patients' compliance [28] and is paid attention in the European Physiotherapy Guideline for PD (EPGPD) [9].

The results of the cluster analysis showed that there were almost no patients with above average impairment but below average motivation. This means that even though some patients with below average impairment are not motivated for physiotherapy, they will be when their condition worsens. However, the later the training starts, the less efficient it is [9]. This result of our study may therefore be used as a motivational tool for poorly motivated patients with below average impairment. This might be also illustrated by our further finding that the importance of stability and falls increases in patients with above average impairment (*p* = 0.000), and they should be therefore motivated to partake in an early physiotherapy balance programme. Such training has already been shown to be effective [29].

The fact that PTs' interest in PD does not depend on the length of their practice or their knowledge about PD might suggest that the necessary education in PD which is a part of implementation of ParkinsonNet can aim at all PTs. The established correlation of the number of PD patients treated yearly with knowledge about PD supports the need to increase the volume of yearly treated patients to gain expertise [3]. Lack of group therapy sessions in our study cohort is probably based on local habitual practice and their implementation might be a way to increase the volume of patients treated yearly as well as to make the therapy more entertaining, thus promoting long-term adherence [9].

The surprising lack of correlation between attendance of a PD specific physiotherapy course with increased knowledge about PD or length and total number of therapy, is given by the fact that there is in fact no such course in Czechia. Those PTs who claimed to have attended such a course actually referred to either the Bobath concept course (which focuses on stroke rehabilitation) or their pregraduate studies.

No correlation of length and total number of therapy with the PTs' knowledge about physiotherapy in PD suggests that the median of 30 minutes of a therapy session repeated eight times was mainly given by custom and not by the PTs' lack of knowledge about the continuum of care recommended by the EPGPD [9]. In theory, the Czech healthcare system has no limits regarding the number and length of physiotherapy sessions, if therapy is reasonably prescribed by a physician (regardless of specialization) and such therapy is generally fully covered by the health insurance, which is obligatory in Czechia. On the other hand, physicians risk in the worst case obligation to pay for the prescribed therapy from their own budget, if they overly exceed the prescription rate from previous years. From this perspective, the Czech healthcare system is at least demotivating. A further explanation might be insufficient knowledge of the physicians about the necessary amount and length of physiotherapy sessions. The EPGPD may provide a useful educational tool as it also entails information for clinicians with detailed description of physiotherapy referral [9].

The most commonly used physiotherapy techniques (NDTs) are not explicitly mentioned anywhere in EPGPD [9]. Based on the definition of conventional therapy [9], NDTs might be implicitly considered a part of it. Nevertheless, strong GRADE-based recommendations for using conventional physiotherapy are based on studies that did not use NDTs but other types of intervention [9]. Moreover, some impairments such as freezing of gait, balance performance, and quality of life have weak recommendation against using conventional physiotherapy to improve them. This suggests that NDTs should not be the most widely used physiotherapy techniques in PD. Similarly, the 3rd most widely used technique, that is, soft tissue or neuromuscular therapy, has only weak recommendation to improve patient-based treatment effect [9]. Other techniques used, that is, gait and balance training, aerobic and resistant training, cueing, and respiratory physiotherapy, were used appropriately [9], but too rarely. Other types of intervention recommended by EPGPD [9] were not mentioned by any of the PTs. The fact that techniques used by PTs did not correlate with knowledge about PD specific physiotherapy (*p* = 0.063) is surprising and probably shows their arbitrary or customary choice. It is therefore again necessary to start evidence-based education in Czechia, which is an integral part of the ParkinsonNet project [3].

Differences in priorities of core areas as reported by patients, PTs, and referring physicians points to the need of better communication between both healthcare professionals and their patients. Even though PTs reported satisfaction with the communication in their team, the quality of this communication can be doubted. Other studies [18, 19] also reported that referring physicians lack information about the benefits of physiotherapy in PD.

The most important barriers for optimal care in Czechia as reported by PTs were few patients treated yearly, insufficient communication, absence of specialized physiotherapy course, and absence of guidelines. These reasons are in accord with previously published Dutch studies [18, 19]. This suggests that implementation of ParkinsonNet should be effective also in Czechia as it was originally designed to overcome these barriers [3] and also proven to be effective in it [16].

## 5. Conclusions

Our data suggest that the present Czech healthcare model for PD patients is suboptimal (low PT prescription rate, small number of patients treated yearly, non-evidence-based physiotherapy, and insufficient communication between healthcare professionals and PD patients). Implementation of EPGPD and the introduction of an efficient model of care such as ParkinsonNet are needed to improve the awareness of the referring neurologists of the benefits of physiotherapy in PD, the prescription rate, and number of PD patients treated yearly by PTs.

## Figures and Tables

**Table 1:** Selected data from the PD patients and PT's questionnaires.

	Gait	Transfers	Manual dexterity	Stability and falls	Posture	Physical condition
Importance (patient opinion): average order (*n* = 248)	1.91	3.35	3.71	3.56	4.43	3.99
Importance (PT opinion): average order (*n* = 91)	2.22	2.95	4.13	2.25	4.85	4.60
Most frequent reasons for referral (*n* = 92)	25% (*n* = 58)	3.45% (*n* = 8)	6.03% (*n* = 14)	27.59% (*n* = 64)	16.38% (*n* = 38)	10.78% (*n* = 25)
Limitations	*n* = 248	*n* = 248	*n* = 248	*n* = 248	*n* = 248	*n* = 248
(i) No difficulties	(i) 4.8% (*n* = 12)	(i) 14.1% (*n* = 35)	(i) 8.5% (*n* = 21)	(i) 17.7% (*n* = 44)	(i) 10.1% (*n* = 25)	(i) 10.1% (*n* = 25)
(ii) Mild difficulties	(ii) 24.6% (*n* = 61)	(ii) 22.6% (*n* = 56)	(ii) 31.0% (*n* = 77)	(ii) 35.1% (*n* = 87)	(ii) 31.0% (*n* = 77)	(ii) 23.4% (*n* = 58)
(iii) Moderate difficulties	(iii) 37.5% (*n* = 93)	(iii) 36.3% (*n* = 90)	(iii) 36.7% (*n* = 91)	(iii) 19.0% (*n* = 47)	(iii) 35.5% (*n* = 88)	(iii) 35.9% (*n* = 89)
(iv) Severe difficulties	(iv) 21.4% (*n* = 53)	(iv) 19.3% (*n* = 48)	(iv) 17.3% (*n* = 43)	(iv) 16.9% (*n* = 42)	(iv) 18.6% (*n* = 46)	(iv) 18.5% (*n* = 46)
(v) Extreme difficulties	(v) 11.7% (*n* = 29)	(v) 7.7% (*n* = 19)	(v) 6.5% (*n* = 16)	(v) 11.3% (*n* = 28)	(v) 4.8 (*n* = 12)	(v) 12.1% (*n* = 30)
Motivation to improve	*n* = 248	*n* = 248	*n* = 247	*n* = 248	*n* = 248	*n* = 248
(i) No interest	(i) 2.8% (*n* = 7)	(i) 5.6% (*n* = 14)	(i) 2.0% (*n* = 5)	(i) 7.3% (*n* = 18)	(i) 6.4% (*n* = 16)	(i) 3.6% (*n* = 9)
(ii) Slight interest	(ii) 0.4% (*n* = 1)	(ii) 1.2% (*n* = 3)	(ii) 0.8% (*n* = 2)	(ii) 0.8% (*n* = 2)	(ii) 1.6% (*n* = 4)	(ii) 0.4% (*n* = 1)
(iii) Neutral	(iii) 12.1% (*n* = 30)	(iii) 11.7% (*n* = 29)	(iii) 13.8% (*n* = 34)	(iii) 10.9% (*n* = 27)	(iii) 9.7% (*n* = 24)	(iii) 9.3% (*n* = 23)
(iv) Rather interested	(iv) 19.8% (*n* = 49)	(iv) 21.8% (*n* = 54)	(iv) 19.0% (*n* = 47)	(iv) 19.7% (*n* = 49)	(iv) 18.2% (*n* = 45)	(iv) 22.6% (*n* = 56)
(v) Interested	(v) 64.9% (*n* = 161)	(v) 59.7% (*n* = 148)	(v) 64.4% (*n* = 159)	(v) 61.3% (*n* = 152)	(v) 64.1% (*n* = 159)	(v) 64.1% (*n* = 159)
Referral to PT	*n* = 248	*n* = 248	*n* = 248	*n* = 248	*n* = 248	*n* = 248
(i) Yes	(i) 20.6% (*n* = 51)	(i) 17.7% (*n* = 44)	(i) 13.7% (*n* = 34)	(i) 15.7% (*n* = 39)	(i) 16.9% (*n* = 42)	(i) 14.1% (*n* = 35)
(ii) No	(ii) 79.4% (*n* = 197)	(ii) 82.3% (*n* = 204)	(ii) 86.3% (*n* = 214)	(ii) 84.3% (*n* = 209)	(ii) 83.1% (*n* = 206)	(ii) 85.9% (*n* = 213)
